# Fecal Indicator Bacteria Data to Characterize Drinking Water Quality in Low-Resource Settings: Summary of Current Practices and Recommendations for Improving Validity

**DOI:** 10.3390/ijerph18052353

**Published:** 2021-02-28

**Authors:** Mustafa Sikder, Elena N. Naumova, Anthonia O. Ogudipe, Mateo Gomez, Daniele Lantagne

**Affiliations:** 1 Department of International Health, Johns Hopkins Bloomberg School of Public Health, Baltimore, MD 21205, USA; 2 Department of Civil and Environmental Engineering, School of Engineering, Tufts University, Medford, MA 02155, USA; elena.naumova@tufts.edu (E.N.N.); Anthonia.Ogudipe@tufts.edu (A.O.O.); mateogomez98@gmail.com (M.G.); daniele.lantagne@tufts.edu (D.L.); 3 Division of Nutrition Epidemiology and Data Science, Friedman School of Nutrition Science and Policy, Boston, MA 02111, USA

**Keywords:** fecal indicator bacteria, membrane filtration, drinking water, LMICs

## Abstract

Fecal indicator bacteria (FIB) values are widely used to assess microbial contamination in drinking water and to advance the modeling of infectious disease risks. The membrane filtration (MF) testing technique for FIB is widely adapted for use in low- and middle-income countries (LMICs). We conducted a systematic literature review on the use of MF-based FIB data in LMICs and summarized statistical methods from 172 articles. We then applied the commonly used statistical methods from the review on publicly available datasets to illustrate how data analysis methods affect FIB results and interpretation. Our findings indicate that standard methods for processing samples are not widely reported, the selection of statistical tests is rarely justified, and, depending on the application, statistical methods can change risk perception and present misleading results. These results raise concerns about the validity of FIB data collection, analysis, and presentation in LMICs. To improve evidence quality, we propose a FIB data reporting checklist to use as a reminder for researchers and practitioners.

## 1. Introduction

Assessing microbial contamination in drinking water is crucial to verify water safety, understand baseline conditions, validate preventive interventions, and investigate disease outbreaks [1]. Fecal indicator bacteria (FIB) values are widely used to characterize microbial contamination [2,3], and there are multiple ways to assess FIB presence and concentration. These include presence/absence, most probable number (MPN), and colony count methods (e.g., membrane filtration, plating, or gel) [2,4,5]. The membrane filtration method is considered a gold standard in quantitative FIB testing and recommended by the American Public Health Association (APHA), American Water Works Association (AWWA), and Water Environment Federation (WEF) in Standard Methods for the Examination of Water and Wastewater [6]. 

To ensure the validity and reproducibility (i.e., replicable sampling and testing protocol) of the membrane filtration test results, step-by-step instructions are available, in Standard Methods and other guidelines [4,6,7]. The instructions focus primarily on sample collection steps and precautions, preservation and storage, laboratory quality control (e.g., personnel, facility, equipment, supply), media preparation, analytical quality control (e.g., plate counts comparison, control culture, duplicate analysis, sterility checks), data handling, and documentation and record-keeping. 

There are specific recommendations about the collection and analysis of water samples for membrane filtration [4,6,7]. It is recommended to complete multiple plates of appropriate serial dilutions for each sample, depending on prior FIB contamination knowledge, source type, and turbidity. The appropriate volume of sample water is passed through a filter paper (mean pore diameter 0.45 μm) using a stand, cup, and pump or syringe. The filter paper is placed on a growth-medium-soaked pad in a Petri dish and incubated at the recommended temperature and duration depending on the media. FIB colonies are manually counted and reported in colony-forming units (CFU) per 100 mL [4,6,7]. It is recommended to complete sample duplicates to quantify precision and blanks to confirm that contamination was not introduced during sample processing [4,6,7]. Please note that we refer to the APHA/AWWA/WEF [6], Centers for Disease Control and Prevention (CDC) [4], and Environmental Protection Agency (EPA) [7]-suggested membrane filter guidelines as “recommended methods” in this manuscript.

While membrane filtration is intended to be completed in a laboratory with dedicated bench space, adequate ventilation, disinfected walls and floors, and necessary equipment [8], it can also be used and adapted for use outside the formal laboratory setting using field test kits [4]. The membrane filtration test method is commonly used to assess microbial contamination in drinking water in low- and middle-income countries (LMICs), where onsite laboratories are not, or may not be, available and researchers bring in their own equipment to test water samples. In LMICs, recommended methods are often adapted depending on the availability of resources, such as being in an informal laboratory setting without access to electricity, autoclaves for sterilization, and refrigerators [2,4]. Typically, the growth medium used during incubation dictates the ideal FIB colony enumeration range (e.g., 20–80) and the maximum number of colonies per membrane above which the petri dish should be assigned to “too numerous to count (TNTC)” [6,9]. In this manuscript, we explicitly focused on FIB data from LMICs because, typically, in high-income settings, formal water quality testing laboratories are easily accessible.

After sample collection and processing, FIB data are prepared for statistical analysis. Typically, field FIB data are skewed, contain outliers and missing data, and are censored by detection limits [10,11,12]. Therefore, appropriate techniques are applied to prepare the data for further analysis. FIB data can be analyzed as continuous, categorical, or binary variables depending on data properties and research objectives [13]. After data preparation, data analysis may include descriptive statistical methods, studying relationships between FIB and relevant factors, and applying regression models. 

FIB data are regularly used to study microbial water quality, measure compliance with guidelines, and evaluate intervention effectiveness [1,14,15,16,17,18]. Relevant examples where relationships are studied include FIB data from different samples (e.g., source, household, along the water chain) [18,19,20,21], with disinfectant and contaminant concentrations (e.g., physical or chemical) [22,23,24], microorganisms (e.g., virus, protozoa) [24,25,26], waterborne disease incidence and prevalence (e.g., diarrhea, cholera) [27], climatic measurements (e.g., temperature, rainfall) [28,29], water, sanitation, and hygiene (WASH) facilities (e.g., toilet, improved water source) [30,31], and user’s behavior (e.g., water collection, transportation, storage, and consumption practices) [32,33]. 

Many researchers expressed their concerns with adapting recommended methods for research in LMICs, including (1) reporting of adherence and adaptation to recommended methods [2], (2) method of sample processing [11,12,34], and (3) data preparation and use of appropriate statistical method for analysis [35,36]. To our knowledge, a summary of membrane filtration practices in LMICs has not been completed to date. Thus, the two objectives of the work presented herein were to (1) synthesize the methods of water sample collection and processing for membrane filtration FIB test and FIB data preparation and analysis, and (2) demonstrate how different data preparation and analysis techniques can impact FIB data analysis results and their interpretation. 

## 2. Materials and Methods

The study consisted of two investigations: (1) a systematic review of FIB data reporting in the published literature, and (2) analysis of selected example FIB datasets to demonstrate how FIB data presentation and analysis impact results. 

### 2.1. Systematic Review of FIB Data Reporting

We completed a systematic review to identify how FIB data are currently collected, analyzed, and reported in the published literature including the development of (1) search strategy, (2) inclusion criteria, (3) selection and processing strategy, and (4) result synthesis. Each step of this systematic review process is summarized below.

### 2.2. Search Strategy

**Figure 1:**
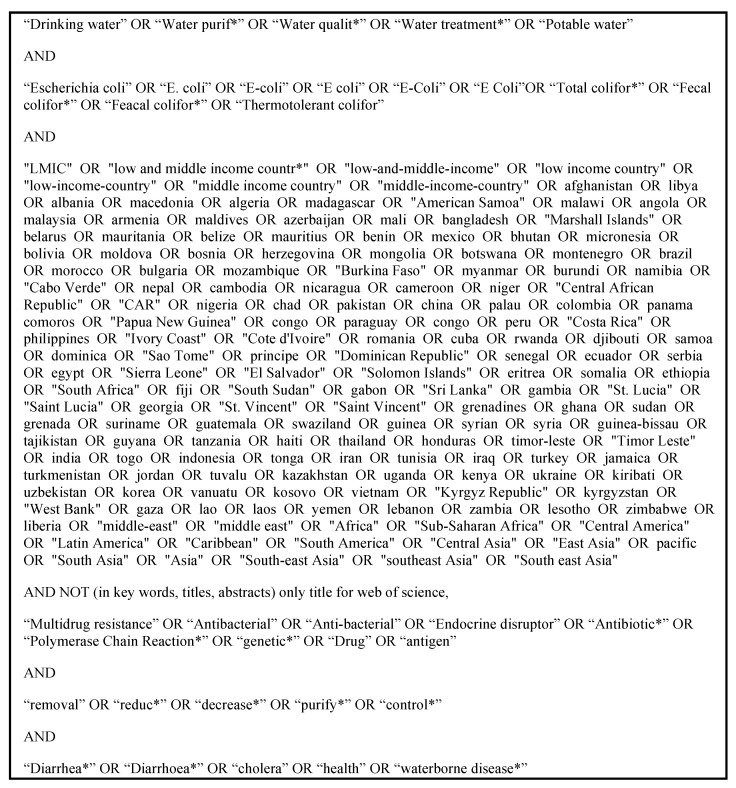
Systematic review search terms (asterisks * indicate search term truncation.).

The databases Ovid Medline (PubMed), Scopus, and Web of Science were searched using a set of search terms related to three themes: low- and middle-income countries (LMICs), FIB, and drinking water, excluding pharmaceutical and agricultural terms (Figure 1). Individualized search strings were developed for each database using appropriate field tags and Boolean operators. We finalized the search in July 2020 to include papers published up to this date.

### 2.3. Inclusion Criteria

Inclusion criteria were developed following the populations, interventions, comparisons, outcomes, and study types (PICOS), adapted for laboratory datasets [37]. The population for this review consisted of FIB test results collected from source or household drinking water samples in LMICs, as defined by the World Bank Income groups in 2018 [38]. To be included, FIB had to be analyzed with the membrane filtration method for total coliform, thermotolerant (fecal) coliform, or *Escherichia coli*; we limited FIB search to the three coliform types because membrane filtration is generally recommended for those three groupings of bacteria [6]. No interventions or comparisons were required for inclusion. Manuscripts were included if the outcome of quantitative analysis of FIB was reported. All study types (i.e., observational and experimental) were eligible for inclusion. Review documents were not included, but individual references in review documents were screened for inclusion. Manuscripts published in English between 1 January 2000 and 25 July 2020 were included in the review. The literature review is reported following the Preferred Reporting Items for Systematic Reviews and Meta-Analyses (PRISMA) guidelines [39]. 

### 2.4. Selection and Processing 

Search results were merged, and the duplicates were removed using EndNote X8.1 (Philadelphia, PA, USA). Unique articles were then screened by title, abstract, and full text using exclusion criteria in Microsoft Excel for Office 365 (Redmond, WA, USA). At title screening, manuscripts not in LMICs or not with source and household water samples were excluded. At abstract screening, in addition to those from title screening, non-membrane filtration FIB testing methods and non-drinking water samples were excluded. In full-text screening, only manuscripts that reported quantitative results from membrane filtration FIB testing of source or household drinking water samples in LMICs were included. 

Studies were independently double-screened by a team of four research assistants and the primary study author. Data were extracted from included studies in a detailed coding sheet that included title, journal, year of publication, abstract, digital object identifier, study type, main objective, description of the sample collection, membrane filtration and bacterial enumeration steps, data preparation, presentation, and statistical techniques applied to the data. Results from independent readers were matched, and differences were resolved by consulting with authors. 

### 2.5. Result Synthesis

Results were synthesized initially by two broad categories: (1) sample collection and processing, and (2) data preparation and analysis. Within each broad category, results were summarized using percentage of manuscripts that included a particular step by sub-category, including, for sample collection and processing, whether manuscripts referenced a standard method, sample collection procedures, and membrane filtration procedures. Please note that steps were identified using Standard Methods. For data preparation and analysis, subcategories included how data were prepared, characterized using descriptive statistics and visualization, and analyzed using statistical methods for correlations and associations. Please note that we did not complete meta-analysis on the data, as that was not necessary for this research and has been completed elsewhere [15]. Additionally, a risk of bias assessment was not completed for each included manuscript, as part of the research question was to determine biases present in the data. 

### 2.6. Analysis of Selected Example FIB Data Sets

To elucidate the impact of various methods for sample collection and processing and data analysis of FIB data identified in the systematic review, we used two publicly available FIB datasets: the 2012–2013 Bangladesh and 2014–2015 Congo Multiple Indicator Cluster Surveys (MICS) [20,40]. In the Bangladesh survey, data were collected between December 2012 and April 2013 from urban and rural areas of seven administrative divisions, covering 20,903 households. In the Congo survey, data were collected between November 2014 and February 2015 from urban and rural areas of 12 administrative departments, covering 12,811 households. MICS datasets were selected for this analysis because of their large sample size representing the full country and to avoid any potential bias by using other secondary data which were collected for a different purpose (i.e., intervention effectiveness or performance evaluation). Congo and Bangladesh surveys were selected because these were the first two national MICS surveys with FIB data completed in South Asia and Africa.

During both surveys, a microbial water quality test was completed for some, but not all, surveyed households and associated water sources. In Bangladesh, 2582 (5%) households and 2532 sources were tested; in Congo, 1486 (12%) households and 1277 sources were tested. Water sample collection and testing procedures were the same in both countries and are fully described in respective reports [40,41]. In summary, household samples of 100 mL were collected by asking for “a glass of water that members of the household would drink” and source samples were collected directly from the source by asking “is it possible to visit the water source from where the drinking water was collected?”, and then enumerators walked to that source after the survey. Enumerators filtered the 100 mL sample through a 0.45 micron filter paper and placed that filter on Compact Dry EC growth medium plates (Nissui, Japan). Separately, 1 mL from the sample was pipetted onto a different Compact Dry EC plate. Plates were incubated in ambient temperature or using incubation belts for 24 h, after which the number of red/purple and blue colonies were recorded. Plates with no colonies were reported as 0, and plates with 100 or more colonies were reported as 100. Please note that the exact location where tests were processed was not specified for each household. However, MICS guidelines recommend processing samples on site if possible or processing in a convenient location by collecting water in WhirlPak^®^ bags (Nasco, Fort Atkinson, WI, USA). If the transportation time was >30 min, samples were placed in a cooler with ice [42]. 

Raw data were downloaded in comma-separated values (CSV) file format from the MICS United Nations International Children’s Emergency Fund (UNICEF) website (https://mics.unicef.org/, accessed on 25 February 2021). Both datasets contained *E. coli* (blue colonies) and non-*E. coli* coliform (red colonies) results in household and corresponding source samples in CFU for 1 mL and 100 mL dilutions. We prepared data for analysis by aggregating results as follows: (1) removing data where the 100 mL sample was 0 and 1 mL was >0 (considered an unreliable result; Bangladesh 3%, Congo 15%); (2) when both values were >0, calculating the geometric mean of 100 mL count value and 100 × 1 mL count value; (3) including the 100 mL count value directly when the 1 mL value was 0. 

Based on the systematic review results, we then completed five analyses on both the Bangladesh and the Congo datasets, to assess the impact of different data replacement, descriptive statistic calculations, visualizations, hypothesis test, and correlation test methods. 

We applied three different data replacement methods on Bangladesh and Congo household *E. coli* data to show the impact of data replacement method on data distribution. The three scenarios were as follows: (*a*) removing below detection limit (BDL) and above detection limit (ADL) samples; (*b*) replacing BDL and ADL samples with the detection limit (i.e., BDL = 0 CFU/100 mL and ADL = 100 CFU/100 mL or 1000 CFU/100 mL); (*c*) replacing BDL values of 0 counts with 0.5 CFU/100 mL, and ADL values (of 100 or 1.00 count value) by adding 1 to the detection limit (i.e., 101 or 1001 CFU/100 mL). We compared the distributions of the log-transformed data using Wilcoxon signed rank tests and Student’s *t*-test. We used the scenario *c* for further analysis. 

To demonstrate the importance of reporting adequate descriptive statistics, we described the household *E. coli* datasets for Bangladesh and Congo grouped by urban and rural areas using 11 parameters (arithmetic mean, geometric mean, standard deviation, geometric standard deviation, median, 25th and 75th percentiles, interquartile range, 10th and 90th percentiles, minimum and maximum, skewness, and kurtosis). 

Additionally, to display the usefulness of visualization techniques, we presented the household *E. coli* Bangladesh and Congo data using a bar plot, boxplot, scatter plot (together with source *E. coli*), and map (the data were grouped by the 12 departments of Congo and seven divisions of Bangladesh, except for the scatter plot). We selected the four visualization techniques because they were commonly used in the reviewed articles. 

To present the importance of hypothesis test methods, we compared Bangladesh household *E. coli* concentration of two districts and Congo household *E. coli* concentration of two divisions using Wilcoxon rank sum test and chi-squared test. We selected two methods appropriate for continuous data and binary variables. The independent observation and random sampling assumptions of Wilcoxon and chi-squared tests were met because each observation was from an independent household and the MICS surveys were designed to randomly select households form the population.

The effect of correlation method selection was demonstrated using Bangladesh and Congo household *E. coli* and coliform (non-*E. coli*) data. We applied Pearson and Spearman correlation methods on the dataset to demonstrate the change in correlation coefficients. The same methods were applied to the log-transformed data to demonstrate the effect of data transformation on the skewed dataset. All data processing and analysis steps were completed in R (Vienna, Austria) [43]. 

Lastly, on the basis of our results, we developed a checklist to consider when membrane filtration-based FIB data are used and reported. The “sample collection” and “membrane filtration” sections of the checklist included critical steps following the recommended methods to understand the field procedure and any adaptation from the guidelines. The “enumeration” and “statistical analysis” sections of the checklist included critical data preparation and statistical steps that will support researchers to communicate the results and readers to understand the findings.

## 3. Results

### 3.1. Systematic Review Results

**Figure 2:**
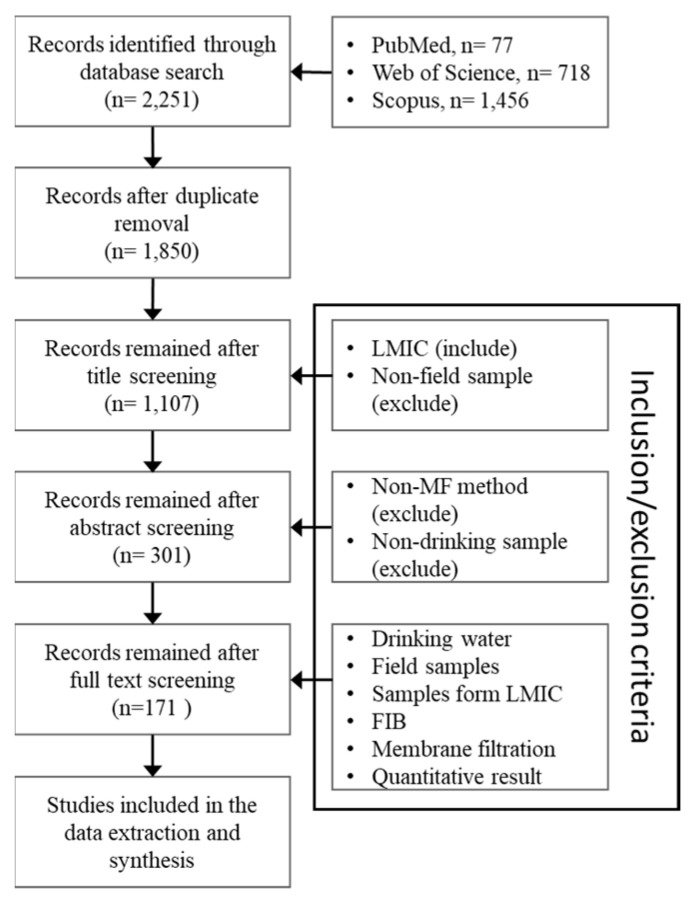
Results of the systematic review screening steps.

To complete our study objectives, we conducted a systematic literature review. Overall, 2251 manuscripts with FIB data in LMICs were identified in the initial and follow-up searches, 1850 unique articles remained after removing duplicates, 1107 were included after title screening, 301 were included after abstract screening, and 171 were included for data extraction after full-text review (Figure 2). The final set of manuscripts represented studies from 48 LMICs. The five most represented countries were Bangladesh in 22, India in 14, Kenya in 12, Cambodia in 11, and South Africa in nine manuscripts. 

#### 3.1.1. Sample Collection and Processing

A total of 95 (56%) manuscripts included a reference to a standard method that was followed for data collection and analysis; the most common referenced method was APHA/AWWA/WEF Standard Methods 58 (34%) (Table 1). Concurrently, 76 (44%) did not reference a standard method (e.g., recommended methods, manufacturer guidance, or published literature). Please note that sometimes manuscript authors referred to following Standard Methods and did not include any other sampling details in the manuscript. 

Standard Methods suggest five key steps for sample collection and transport: (1) collect in sterile glass or plastic; (2) use sodium thiosulfate (to inactivate any chlorine or bromine present and prevent ongoing disinfection); (3) collect a representative sample from the source; (4) if not analyzed within 1 h, place on ice and maintain temperature of <10 °C; (5) for drinking water samples, begin analysis within 6 h of collection and, for non-drinking water samples, begin analysis within 24 h. A total of 66 (39%) studies reported any sample collection sterility information (e.g., sterile vial/bag, hand sanitization before collection), 34 (20%) studies reported using sodium thiosulfate, 102 (60%) reported storing the sample at “low” temperature (from 2–8 °C), and 94 (55%) reported the time between sample collection and membrane filtration (2–48 h). Of the 94 reporting the time, 50 (53%) met the criteria of analysis begun within 6 h of collection and completed within 8 h.

Standard Methods suggest that, in membrane filtration, sterile apparatus should be used, positive and negative controls at the beginning and end of sampling should be completed, 10% of the plates should be duplicated, 5% should be blank, appropriate dilutions based on water quality should be selected, samples should be filtered and placed on a selective medium-soaked pad, and then those samples should be incubated at an appropriate temperature for the appropriate time. In systematic review results (Table 1), 31 (18%) reported using blank samples (negative controls) to check sterile procedures, 44 (26%) reported using duplicate samples to check analysis precision, 41 (24%) reported using multiple appropriate dilutions based on water source, 80 (47%) reported the volume of filtered sample water, 126 (74%) reported the name/type of the growth medium, 112 (65%) reported incubation temperature, and 110 (64%) reported incubation time.

#### 3.1.2. Data Preparation and Analysis

Standard Methods do not provide specific analysis techniques, but recommend discarding data if controls are contaminated, only counting plates where a certain number of colonies have grown (dependent on media; e.g., 20–80 colonies, and no more than 200–250), only including in analysis “countable” plates, reporting BDL and ADL results, and that data are likely to be skewed and should be log-transformed. Please note that ADL samples are referred to as too numerous to count (TNTC) in FIB reporting. Regarding data preparation, 82 (48%) manuscripts reported using >1 dilution, 35 (20%) manuscripts reported how values from multiple dilutions were aggregated, 84 (49%) reported the percent of BDL and ADL samples, 46 (27%) reported how BDL results were handled, 49 (29%) reported how ADL results were handled, and 73 (43%) studies reported log-transforming data (Table 1).

In the review, we identified four primary FIB data analysis objectives: characterize results (171, 100%) using descriptive statistics (136, 80%) and/or data visualization (87, 51%), test a hypothesis by comparing results between groups or against standards (45, 26%), study associations between FIB and other variables (60, 35%), and conduct regression analyses (44, 26%). Details of the assessment are presented below.

Frequently reported descriptive statistics were proportion/frequency (85, 50%), arithmetic mean (74, 43%), and minimum/maximum range (53, 31%). Additionally, 95% confidence interval of the mean was reported in 44 (26%), median in 36 (22%), standard deviation in 38 (21%), geometric mean in 32 (22%), percentile in 15 (9%), and interquartile range in 14 (8%) manuscripts. No manuscript reported skewness and kurtosis of the data. Overall, 75 (45%) manuscripts reported log-transforming the data.

Additionally, 87 (51%) studies used at least one data visualization technique to report FIB data. The most commonly used visualization methods were bar or column graph (50, 29%), followed by box plot (21, 12%), time-series plot (17, 10%), scatter plot (13, 8%), and maps (7, 4%).

To compare to standards, 50 (29%) studies converted data into categorical data and categorized data according to the World Health Organization (WHO)’s risk categories [1], and 45 (26%) studies converted the data into binary data to compare to WHO’s guideline value of <1 FIB/100 mL [1] or secondary guideline of “intermediate risk” at <10 CFU/100 mL [1]. In the data, 40 (23%) studies used the 1 CFU/100 mL cutoff value, and five (3%) studies used 10 CFU/100 mL.

To compare to other groups of data, manuscripts used parametric and nonparametric tests. The most reported (*n* = 39, 23%) comparison method was Student’s *t*-test, followed by chi-squared test (25, 15%), ANOVA (24, 14%), Wilcoxon rank-sum test (20, 12%), Fisher’s exact test (20, 12%), Kruskal–Wallis test (14, 8%), Wilcoxon signed-rank test (9, 5%), and McNemar’s test (4, 2%). Of those (*n* = 63) who reported using parametric tests, only five (8%) studies reported completing any data normality assumption check (e.g., Shapiro–Wilk test, quantile–quantile (QQ) plot, histogram).

In assessing association methods, we found that 18 (11%) manuscripts reported the use of Pearson correlation coefficients, and 12 (7%) studies reported the use of Spearman correlation coefficients. Odds ratios were reported by 24 (14%) studies and risk ratio was reported by 10 (6%) studies. Please note that the use of advanced statistical techniques (e.g., multivariate regression models) was outside the scope of this review.

### 3.2. Analyses Using Example Dataset

According to the systematic review data, we demonstrate the use of five common FIB data analysis methods for the publicly available Congo and Bangladesh FIB datasets. We assessed the utilization of different data replacement methods, descriptive statistic calculations, visualization tools, hypothesis/comparison test methods, and correlation test methods.

#### 3.2.1. Data Replacement Methods

**Figure 3:**
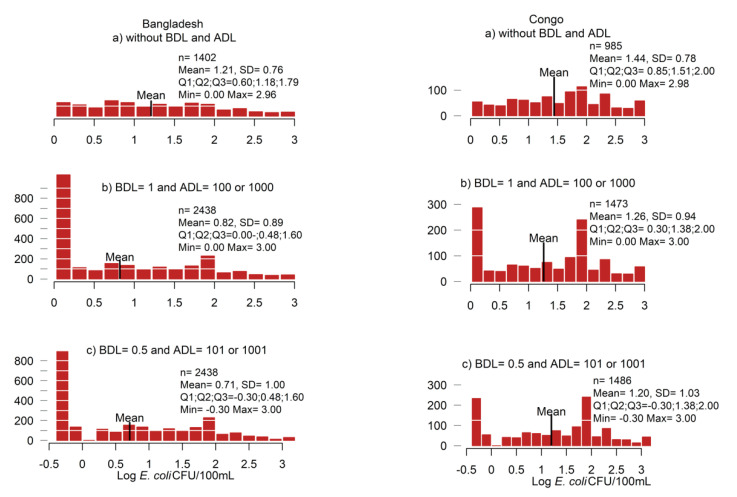
Characterization of *Escherichia coli* data from Bangladesh (**left column**) and Congo (**right column**) household water samples applying three BDL and ADL replacement methods: (**a**) excluding ADL and BDL data; (**b**) replacing BDL = 1 and ADL = 100 or 1000 depending on the sample volume; (**c**) replacing BDL = 0.5 and ADL = 101 or 1001 depending on sample value.

To document any impact on results of different BDL and ADL replacement methods, household *Escherichia coli* (*E. coli*) CFU/100 mL datasets from Bangladesh and Congo were analyzed. According to recommendations from Standard Methods [44] and what was reported in the systematic review, we prepared data using three methods: (a) removed censored data; (b) replaced BDL and ADL with the detection limit; (c) replaced BDL with 0.5 and ADL with adding 1 to the detection limit. Histograms of log-transformed data are presented in Figure 3.

As can be seen, the form of FIB distributions changed depending on BDL/ADL replacement method. All pairwise comparisons were statistically significantly different (all Wilcoxon signed rank test, *p* < 0.001). Means of log-transformed values were also significantly different (all *t*-test, *p* < 0.001) between the BDL/ADL replacement methods. The mean values were highest for method *a* (Bangladesh: 1.21, Congo: 1.44) and smallest for method *c* (Bangladesh: 0.71, Congo: 1.2). Additionally, the distribution spread was largest for method *c* (standard deviation (SD): 1.00 and 1.03 and interquartile range (IQR): 1.9 and 2.3 for Bangladesh and Congo) and smallest for method *a* (SD: 0.76 and 0.78 and IQR: 1.19 and 1.15 for Bangladesh and Congo).

#### 3.2.2. Descriptive Statistics

In Standard Methods, it is recommended to use the geometric mean for estimating central tendency, except in risk assessment, where the arithmetic mean may provide a greater safety factor [8]. It is also noted that the data will be skewed. In the systematic review, the most commonly reported descriptive statistic was the frequency of WHO risk category, and distribution information was rarely reported. While WHO categorization could effectively convey the risk, understanding the data properties is important for further statistical analysis. To demonstrate the effect of descriptive statistics selection, 11 different descriptive statistics were applied to the Bangladesh and Congo FIB datasets stratified by urban and rural areas (Table 2). As shown in Table 2, the selection of a descriptive statistic influences the results. In particular, geometric mean was consistently one WHO risk category [1] below the arithmetic mean.

Reporting additional descriptive statistics can characterize FIB distributions and justify statistical test selection. For example, standard deviation and interquartile range can help understand data variability; as FIB data are generally skewed, reporting 25th, 50th (median), and 75th percentiles is useful in detecting outliers or extreme values which can result from multiplying with dilution factors; refined values for percentiles (e.g., 5th, 10th, 90th, and 95th) are informative to understand data spread; data range (minimum and maximum) presents detection limits of the FIB test; skewness (measure of distribution symmetry) and kurtosis (measure of distribution tail extensions) characterize the extent of deviation of the FIB data from a normal symmetrical distribution. For instance, none of the four groups had skewness and kurtosis values close to 0 and 3 (typical for a normal distribution), respectively. This suggests that the Bangladesh and Congo data did not follow the normal distribution and, thus, parametric tests are likely to be inappropriate and data transformation or nonparametric tests are more suitable for analysis than traditional parametric tests. In fact, the presented results suggested that samples from urban areas had higher FIB concentration than rural areas in Bangladesh and samples from rural areas had higher FIB concentration than urban areas in Congo.

#### 3.2.3. Visualizations

**Figure 4:**
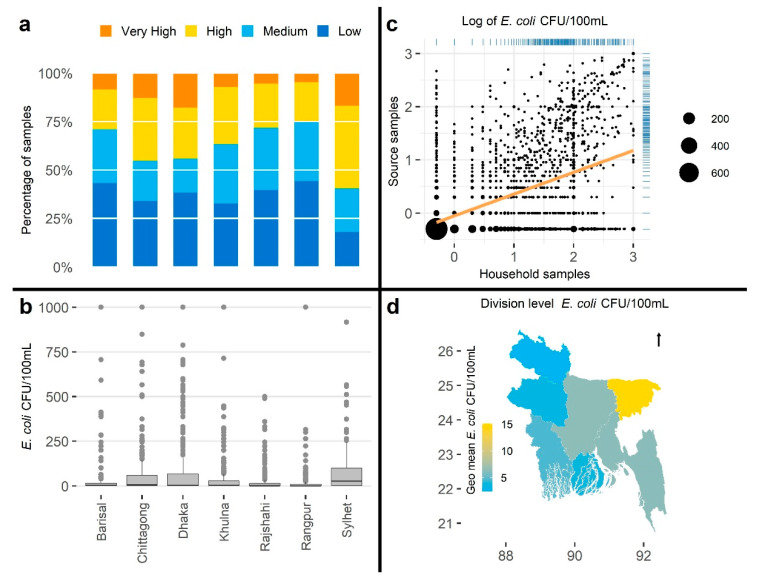
Examples of different visualization for Bangladesh data: (**a**) bar plot of World Health Organization (WHO) risk categories in household water samples by administrative units, (**b**) box plot of *E. coli* colony-forming units (CFU)/100 mL in household water samples by administrative units, (**c**) scatter plot of log-transformed *E. coli* CFU/100 mL in household and corresponding source water samples, and (**d**) map of geometric mean of *E. coli* CFU/100 mL in household water samples by administrative units.

**Figure 5:**
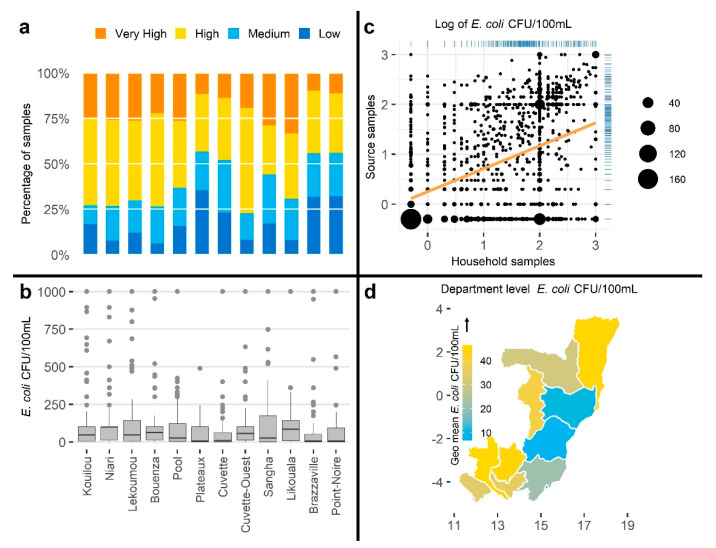
Examples of different visualization for Congo data: (**a**) bar plot of WHO risk categories in household water samples by administrative units, (**b**) box plot of *E. coli* CFU/100 mL in household water samples by administrative units, (**c**) scatter plot of log-transformed *E. coli* CFU/100 mL in household and corresponding source water samples, and (**d**) map of geometric mean of *E. coli* CFU/100 mL in household water samples by administrative units.

As found in the systematic review, data visualizations were provided in slightly over half of the manuscripts. Appropriate FIB data visualization can aid reporting by emphasizing relevant characteristics (e.g., distribution, risk category proportions, spatial and temporal variation, and correlation). To demonstrate the impact of different visualizations on data interpretation, we visualized data from Bangladesh and Congo using four plot types frequently seen in the systematic review: bar chart using WHO risk categories, box plot presenting *E. coli* concentration distribution grouped by administrative units, scatter plot demonstrating correlation between two variables, and maps to communicate spatial variation of the *E. coli* concentration (Figure 4 and Figure 5).

Perception of information provided by different visualization tools could be severely affected when used without understanding plot limitations. For example, if the objective is to present the water quality using WHO risk category, a bar plot illustrating the composites of samples with very high, high, medium, and low concentration by location (Figure 4a and Figure 5a) would be a useful approach. Similarly, a box plot will clearly illustrate the distribution of *E. coli* concentration in the water samples (Figure 4b and Figure 5b), a scatter plot will be useful to present the relationship between two comparable variables (Figure 4c and Figure 5c), and a map will visualize spatial variations of *E. coli* concentrations aggregated by mapping unit (Figure 4d and Figure 5d).

#### 3.2.4. Hypothesis/Comparison Testing

As found in the systematic review, one-quarter of manuscripts reported testing hypothesis via group comparisons. These comparisons can be completed using different data types, e.g., using binary, categorical, or continuous data. In this analysis, we compared FIB concentration between two adjacent administrative divisions in Bangladesh (Rajshahi and Khulna) and two adjacent departments in Congo (Kouilou and Pointe-Noire), using two approaches (Table 3). First, we used the original continuous data and then we concerted continuous values into binary variable by using two cutoffs of ≥1 and ≥10. In Bangladesh data, the result from the Wilcoxon rank sum test (used because the data were not normally distributed) indicated that the *E. coli* levels in household samples were significantly different between the two divisions. However, the chi-squared test applied to binary variable suggested that the *E. coli* levels were not statistically different between the two divisions for either cutoff value (≥1 and ≥10). In the Congo data, the Wilcoxon rank sum test and chi-squared test for cutoff ≥10 suggested that the *E. coli* levels in the household samples were significantly different between the two departments. However, the chi-squared test with cutoff ≥1 suggested that the levels were not statistically different between the two departments.

The method for statistical comparisons should be selected with respect to the research question and statistical properties of the data. For example, if the research question is about detecting the difference between FIB concentration in household samples in different divisions, continuous data may offer a more reliable and consistent inference than data split into categories. However, if the research question is about the difference in the proportion of households with contaminated samples (e.g., FIB values above specific thresholds) between the two divisions, then the binary variable should be used for statistical testing. Additionally, conversion from continuous FIB data to binary using different cutoffs should be completed with caution as the result may change depending on the cutoff threshold, as seen with the Congo data.

#### 3.2.5. Associations

**Figure 6:**
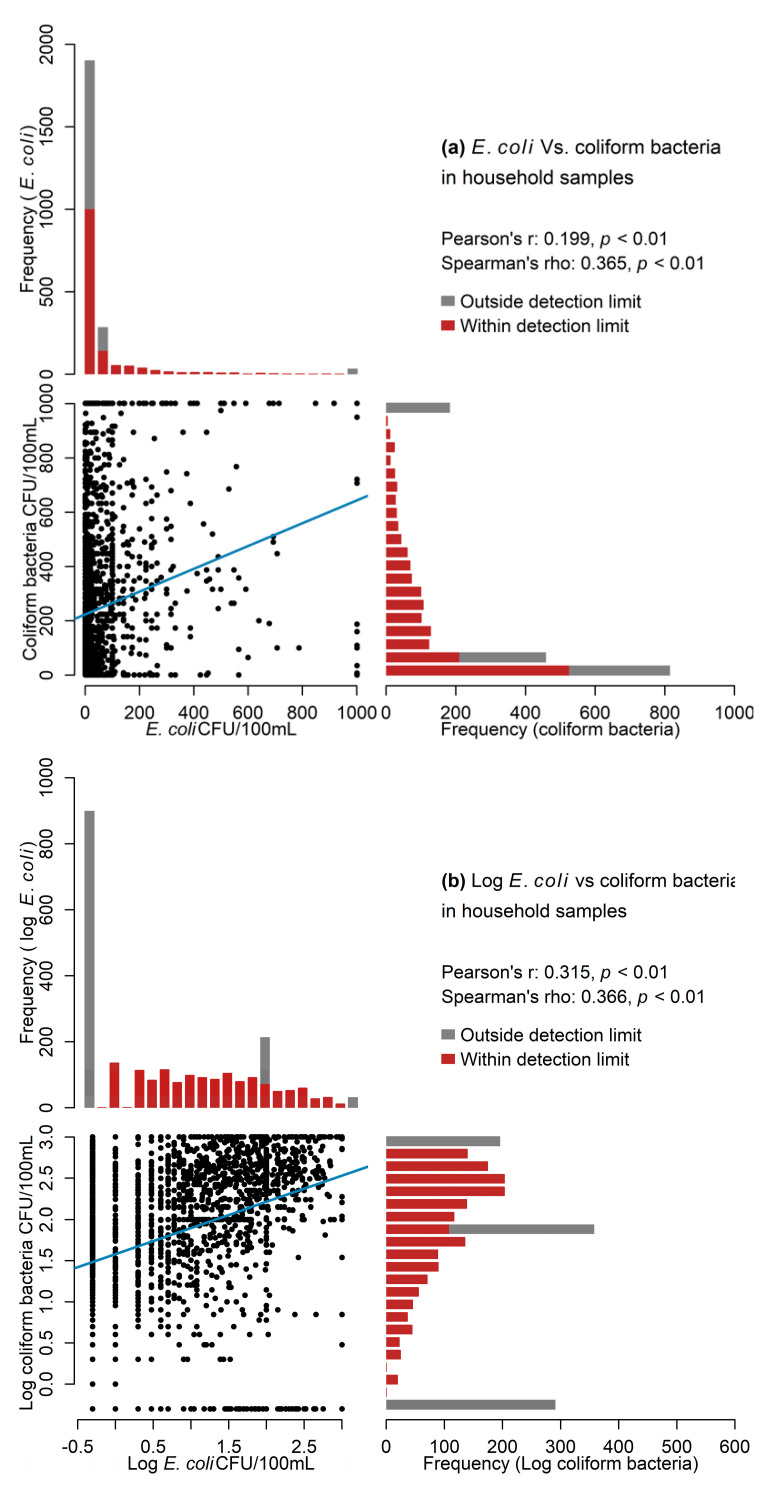
Multipanel plots combining a scatterplot with adjacent histograms to illustrate association between *E. coli* and other coliform values in household water samples, in Bangladesh: (**a**) without transformation; (**b**) log-transformed data.

As seen in the systematic review, associations were tested in 36% of the manuscripts, using Spearman and Pearson correlations. Pearson correlation coefficients are suitable for testing linear associations for variables with distributions that are preferably symmetric and close to normal, whereas Spearman correlation is a good alternative for a monotonic relationship and distributions that are slightly skewed. We demonstrated the effect of choosing different correlation techniques in assessing the associations between *E. coli* and other coliform bacteria concentrations in household water samples in Bangladesh and Congo data (Figure 6) using multipanel plots [45].

It is commonly assumed that total coliform and *E. coli* are correlated [5]. As can be seen (Figure 6), the right-skewed histograms of both variables suggested that the data are non-normal. Pearson correlation between the two variables yielded a weak association (*r* = 0.199, *p*-value < 0.001), while Spearman correlation showed a moderate association (ρ = 0.365, *p*-value < 0.001) (Figure 6) from the Bangladesh data. In the Congo data, Pearson correlation suggested moderate association (*r* = 0.382, *p*-value <0.001), while Spearman correlation showed stronger association (ρ = 0.559, *p*-value <0.001) between the two variables. In this case, Pearson correlation is likely to underestimate the true associations picked up by the Spearman correlation coefficient, because Spearman correlation uses ranked (i.e., relative position label as first, second, third, etc.) values, unlike the Pearson correlation coefficient that utilizes the actual FIB values. Thus, correct magnitude of association may not be observed if the method to detect association is applied without considering FIB data properties, especially as the FIB data are generally not normally distributed.

A different approach to study association when distribution is skewed is to apply Pearson correlation to log-transformed values. For instance, if data are log-transformed, the Pearson correlation (*r*_Bangladesh_ = 0.315, *r*_Congo_ = 0.492) and Spearman correlation (ρ_Bangladesh_ = 0.366, ρ_Congo_ = 0.559) coefficients are close to each other.

## 4. Discussion

To understand how FIB data are produced by membrane filtration in LMICs, we conducted a systematic review of the literature and analyzed publicly available datasets. FIB data are collected and analyzed using membrane filtration, and they are reported frequently by researchers in LMIC contexts. In the systematic review, it was found that sample collection and processing steps were under-reported, and different statistical methods were used to analyze data. Analyzing the publicly available datasets, we demonstrated that different statistical methods can significantly change results and/or interpretation; for example, (1) different data preparation methods can change the FIB data distribution, (2) data description parameters can change the FIB risk perception and communicate misleading information, (3) different hypothesis test methods can produce contrasting results, and (4) different statistical correlation methods can produce different levels of association. We describe each of these findings below and propose a checklist for FIB data reporting, analysis, and presentation.

While there are standard methods for membrane filtration sample collection and processing, reporting adherence to these methods in the published literature was limited. It is recommended to have a sample collection plan that adheres to standards and report that when publishing data. This will increase research reliability. Additionally, there are common adaptations to standard methods used in LMICs, including extending holding times before analysis and storing the sample at low temperature (Table 1). The impacts of these adaptations are not always known, although research has been completed to show limited impact from extending holding time [46] or not having consistent incubation temperature [47], and other research has found significant impact results from not using thiosulfate in sample collection [48]. Further research to determine the impact of commonly used adaptations of membrane filtration for use in LMICs is warranted.

In the systematic review, we found that a variety of statistical techniques were applied to FIB data. With improved data collection and reporting, novel applications of sophisticated analytical methods could advance the use of FIB for in-depth spatiotemporal analysis and modeling [49,50]. However, inadequate data descriptions and frequent use of these tests without proper justification were observed. While the use of well-grounded statistical methods strengthens research inference to report and interpret FIB data, erroneous applications of statistical procedures raise questions about findings and undermine the research validity. Of particular note in the review, (1) while a plethora of literature is available to handle censored environmental datasets [11,34,51,52,53,54], fewer than one-third of the studies reported steps completed to replace BDL and ADL values; (2) descriptive statistics are universally reported in manuscripts, and, while useful to understand FIB concentrations, the prevalence of reporting only one statistic or only the mean is misleading; (3) clear articulation of the research questions and use of appropriate statistical techniques for testing will ensure valid results; relationships between FIB data and other variables are frequently included in evaluations, and results can impact decision-making and policy.

In some instances, statistical analyses that use FIB counts/concentrations rather than risk categories can be problematic. FIB results have inherent uncertainty because of the spatiotemporal variability of bacteria populations, the patchiness/clumping of bacteria in water, the lack of correlation between *E. coli* concentrations and pathogen concentrations [55], and dependence on physical and biological conditions [56,57]. In assessing temporal variability of *E. coli* concentrations, the statistical analysis that treats FIB concentrations as precise is reasonable [52]. However, the use of FIB counts/concentrations to indicate the risk of fecal contamination should be considered with high caution. Furthermore, interpreting statistical results as the precise indication of health risk could be misleading. In such circumstances, categorization (for example, with WHO’s risk categories) replaces the FIB values and shifts the focus on reporting the risk category. Although risk categories can obfuscate the “intuitive differences” between FIB concentrations (for example, between 11 and 99 CFU/100 mL), one of the reasons to use them is to avoid overstating precision or confidence when it is uncertain what the difference between 11 and 99 is actually indicating.

As seen with the data analysis examples presented in the results, inappropriate data processing and analysis methods can result in misinterpretations and erroneous conclusions. This needs to be avoided; thus, it is recommended that, in LMIC settings with limited laboratory support, appropriate selection of and reporting of sample collection and processing and data preparation and analysis should be an integral part of FIB research. Additionally, sharing raw and/or processed data can improve the replicability of the results. To that end, we present a checklist (Table 4) that can be used to develop a sample collection plan and report results.

While membrane filtration is considered the gold standard in certified laboratories, it is challenging to conduct high-quality membrane filtration testing in research field laboratories in LMIC settings. As we found in the review, quality control steps (e.g., duplicates, blanks) are often not reported. Depending on the trained staff availability, resources, equipment, and research questions, alternative methods (such as MPN methods) may provide reliable results [4,58,59]. If membrane filtration is selected, we propose following and reporting the process using the checklist included in Table 4. By improving FIB data reporting, the quality of publication with FIB data will also improve and have a better chance to reach a broader audience [60].

Limitations of the systematic review were as follows: (1) only peer-reviewed articles published in English were included, and (2) only three electronic citation databases were initially searched. While the inclusion of other languages and more databases may have increased the number of articles, we do not feel these limitations impacted results. Limitations in the publicly available datasets were as follows: (1) exact information on field sampling procedure was not available, and (2) all samples were processed for 1 mL and 100 mL dilutions without consideration of water source; this could have produced more BDL and ADL results than when dilutions are carefully considered. However, as the datasets were used to present examples, we considered that using these datasets was a better option to alternatives such as using a simulated dataset. Additionally, the topics outlined in Table 4 should be viewed as indicative and not as an exhaustive list of parameters that will meet the reporting needs for all possible FIB data collection, processing, and analysis scenarios. We propose using the checklist as a preliminary tool to assess the inclusion of relevant information. Determining the impact of sample collection and processing and data preparation on more advanced statistical techniques (e.g., regression, time-series analysis) was outside the scope of this manuscript. Lastly, as future research, we recommend completed detailed investigations of the individual issues identified in this review to establish guidelines for FIB data analysis.

## 5. Conclusions

Membrane filtration methods are commonly used in LMICs to assess drinking water quality risk. Our review results show that, generally, sample collection and processing techniques and data preparation and analysis methods are inadequately reported and can be inappropriate, which, as seen herein, can lead to misleading results. We found limited reporting of adherence and adaptation in using membrane filtration methods. Additionally, using example datasets, we demonstrated the results of different statistical method selection on FIB data analysis results and/or interpretation. Our example analysis highlights the importance of adequate reporting of FIB data usage in LMIC. Lastly, to standardize FIB data collection, processing, and analysis reporting, we proposed a checklist. We hope the topics discussed in this manuscript will assist researchers to strengthen FIB results and assist reviewers and readers in interpreting FIB results in LMICs.

## Figures and Tables

**Table 1:** Literature review results on reporting the sample collection, analysis, and processing. BDL, below detection limit; ADL, above detection limit.

Reported Topics	*N* = 171
Collection	
Included a reference to standard method	95 (56%)
Included any sample collection sterility information	66 (39%)
Used sodium thiosulfate	34 (20%)
Reported storing the sample in “low” temperature (range: 2–8 °C)	102 (60%)
Reported time between sample collection and membrane filtration (range: 2–48 h)	94 (55%)
Reported starting the test in 6 h and/or completed in 8 h	50 (29%)
Analysis	
Reported using blank samples (negative controls) to check sterile procedures	31 (18%)
Reported using duplicate samples to check the precision of the analysis	44 (26%)
Reported using multiple appropriate dilutions based on water source	41 (24%)
Reported the volume of filtered sample water	80 (47%)
Reported the name/type of the growth media	126 (74%)
Reported incubation temperature	112 (65%)
Reported incubation time	110 (64%)
Processing	
Reported how values from multiple dilutions were aggregated	35 (20%)
Reported the percent of BDL and ADL samples	84 (49%)
Reported how BDL results were handled	46 (27%)
Reported how ADL results were handled	49 (29%)
Reported log transforming data	73 (43%)

**Table 2:** Descriptive statistics for fecal indicator bacteria (FIB) concentrations in urban and rural areas for Bangladesh and Congo datasets.

	Bangladesh		Congo	
Category	Urban (*n* = 390)	Rural (*n* = 2048)	Urban (*n* = 503)	Rural (*n* = 970)
Arithmetic mean	63.0	51.7	66.5	124.0
Geometric mean	4.9	5.2	8.0	22.7
Standard deviation (SD)	168.0	136.0	160.8	221.9
Geometric SD	10.8	9.8	10.4	9.9
Median	3	3	9	42
25th and 75th percentiles	0.5–33.8	0.5–40.0	0.5–76.7	0.5–100.0
Interquartile range (IQR)	35.3	39.5	76.2	96.0
10th and 90th percentiles	0.5–141.4	0.5–114.0	0.5–141.4	0.5–316.2
Minimum and maximum	0.5–1001.0	0.5–1001.0	0.5–1001.0	0.5–1001.0
Skewness	4.1	4.9	4.5	2.9
Kurtosis	17.6	27.6	21.7	7.9

**Table 3:** Comparison between FIB concentrations in two administrative units (divisions in Bangladesh and departments in Congo) using binary and continuous variables.

Data Type	Test	Null *H_0_*	Statistic	*p*-Value
Bangladesh				
Continuous	Wilcoxon rank sum test	Medians are equal in both divisions	W = 68,041	0.025 *
Binary (cutoff ≥ 1)	Pearson’s chi-squared test	Proportions are equal in both divisions	χ^2^ = 3.82	0.051
Binary (cutoff ≥ 10)			χ^2^ = 3.79	0.052
Congo				
Continuous	Wilcoxon rank sum test	Medians are equal in both departments	W = 5952	<0.001 *
Binary (cutoff ≥ 1)	Pearson’s chi-squared test	Proportions are equal in both departments	χ^2^ = 3.34	0.068
Binary (cutoff ≥ 10)			χ^2^ = 13.32	<0.001 *

* *p*-Value < 0.05.

**Table 4:** Checklist of recommended parameters to report in manuscripts including membrane filtration data from low- and middle-income countries (LMICs).

Section/Topic	Checklist Item
Sample collection	
1	Report sample collection equipment and supplies (e.g., sterile bottle/bag/vial)
2	Report if sodium thiosulfate (or equivalent) was used (if chlorinated sample)
3	Report if aseptic procedure was maintained to prevent contamination
4	If not analyzed in one hour, report if <10 °C was maintained
5	If not analyzed immediately, report the time between collection and analysis
Membrane filtration	
6	Report if positive and negative controls were checked
7	Report the volume of sample filtered
8	Report number, dilution, and/or volume of serial dilutions
9	What diluent was used if any
10	Report the selective growth media
11	Report the incubation time and temperature
Enumeration	
12	Report the detection minimum/maximum range for enumeration
13	Report aggregation method for serial dilutions
14	Report the number of BDL and ADL samples
15	If BDL/ADL samples were included in analysis, report how values were replaced
16	Report if any data were dropped due to positive/negative controls
17	If a subset of enumerations were verified by a second person
Statistical analysis	
18	Report if the data were transformed
19	Report if the data were analyzed as count, continuous, categorical, and binary
20	Describe dataset using parameters that justify any following statistical analysis
21	For data visualization, ensure proper tool was selected to aid information communication
22	Provide rationales for the choice of statistical method
23	Report if the data met the assumptions of the selected statistical test

## Data Availability

We confirm that all data sets, scripts, and images are available from the corresponding author upon request. The list of articles is available from the corresponding author upon request.

## References

[1] World Health Organization (2017). Guidelines for Drinking-Water Quality: Fourth Edition Incorporating The First Addendum.

[2] Bain R., Bartram J., Elliott M., Matthews R., Mcmahan L., Tung R., Chuang P., Gundry S. (2012). A summary catalogue of microbial drinking water tests for low and medium resource settings. Int. J. Environ. Res. Public Health.

[3] Bain R., Cronk R., Hossain R., Bonjour S., Onda K., Wright J., Yang H., Slaymaker T., Hunter P., Prüss-Ustün A. (2014). Global assessment of exposure to faecal contamination through drinking water based on a systematic review. Trop. Med. Int. Health.

[4] Centers for Disease Control and Prevention (CDC) (2010). Microbiological Indicator Testing in Developing Countries: A Fact Sheet for the Field Practitioner.

[5] Fewtrell L., Bartram J., WHO (2001). Indicators of microbial water quality. Water Quality: Guidelines, Standards and Health.

[6] (2017). APHA/AWWA/WEF 9222 Membrane Filter Technique for Members of the Coliform Group (2017). Standard Methods for the Examination of Water and Wastewater.

[7] Environmental Protection Agency, Office of Water (2002). Method 1604: Total Coliforms and Escherichia coli in Water by Membrane Filtration Using a Simultaneous Detection Technique (MI Medium).

[8] (2018). APHA/AWWA/WEF 9020 Quality Assurance/Quality Control (2017). Standard Methods for the Examination of Water and Wastewater.

[9] Hach Analytical Procedures (1999). m-ColiBlue24 Broth Procedure for Membrane Filtration.

[10] Haas C.N. (1996). How to average microbial densities to characterize risk. Water Res..

[11] Haas C.N., Scheff P.A. (1990). Estimation of averages in truncated samples. Environ. Sci. Technol..

[12] Alexander N. (2012). Analysis of Parasite and Other Skewed Counts. Trop. Med. Int. Health.

[13] Clasen T.F., Alexander K.T., Sinclair D., Boisson S., Peletz R., Chang H.H., Majorin F., Cairncross S. (2015). Interventions to improve water quality for preventing diarrhoea. Cochrane Database Syst. Rev..

[14] Gruber J.S., Ercumen A., Colford J.M. (2014). Coliform bacteria as indicators of diarrheal risk in household drinking water: Systematic review and meta-analysis. PLoS ONE.

[15] Bain R., Cronk R., Wright J., Yang H., Slaymaker T., Bartram J. (2014). Fecal Contamination of Drinking-Water in Low- and Middle-Income Countries: A Systematic Review and Meta-Analysis. PLoS Med..

[16] Hunter P.R. (2003). Drinking water and diarrhoeal disease due to Escherichia coli. J. Water Health.

[17] Williams A.R., Bain R.E.S., Fisher M.B., Cronk R., Kelly E.R., Bartram J. (2015). A systematic review and meta-analysis of fecal contamination and inadequate treatment of packaged water. PLoS ONE.

[18] Wright J., Gundry S., Conroy R. (2004). Household drinking water in developing countries: A systematic review of microbiological contamination between source and point-of-use. Trop. Med. Int. Health.

[19] Hamzah L., Boehm A.B., Davis J., Pickering A.J., Wolfe M., Mureithi M., Harris A. (2020). Ruminant fecal contamination of drinking water introduced post-collection in rural kenyan households. Int. J. Environ. Res. Public Health.

[20] Ercumen A., Naser A.M., Unicomb L., Arnold B.F., Colford J.M., Luby S.P. (2015). Effects of source-versus household contamination of tubewell water on child diarrhea in Rural Bangladesh: A randomized controlled trial. PLoS ONE.

[21] Sikder M., Mirindi P., String G., Lantagne D. (2020). Delivering Drinking Water by Truck in Humanitarian Contexts: Results from Mixed-Methods Evaluations in the Democratic Republic of the Congo and Bangladesh. Environ. Sci. Technol..

[22] Sikder M., String G., Kamal Y., Farrington M., Rahman A.S., Lantagne D. (2020). Effectiveness of water chlorination programs along the emergency-transition-post-emergency continuum: Evaluations of bucket, in-line, and piped water chlorination programs in Cox’s Bazar. Water Res..

[23] Singh A.K., Das S., Singh S., Pradhan N., Gajamer V.R., Kumar S., Lepcha Y.D., Tiwari H.K. (2019). Physicochemical parameters and alarming coliform count of the potable water of Eastern Himalayan state Sikkim: An indication of severe fecal contamination and immediate health risk. Front. Cell Dev. Biol..

[24] Fisher M.B., Williams A.R., Jalloh M.F., Saquee G., Bain R.E.S., Bartram J.K. (2015). Microbiological and chemical quality of packaged sachet water and household stored drinking water in Freetown, Sierra Leone. PLoS ONE.

[25] Affum A.O., Osae S.D., Nyarko B.J.B., Afful S., Fianko J.R., Akiti T.T., Adomako D., Acquaah S.O., Dorleku M., Antoh E. (2015). Total coliforms, arsenic and cadmium exposure through drinking water in the Western Region of Ghana: Application of multivariate statistical technique to groundwater quality. Environ. Monit. Assess..

[26] Wu J., Long S.C., Das D., Dorner S.M. (2011). Are microbial indicators and pathogens correlated? A statistical analysis of 40 years of research. J. Water Health.

[27] Luby S.P., Halder A.K., Huda T.M., Unicomb L., Islam M.S., Arnold B.F., Johnston R.B. (2015). Microbiological contamination of drinking water associated with subsequent child diarrhea. Am. J. Trop. Med. Hyg..

[28] George C.M., Jung D.S., Saif-Ur-Rahman K.M., Monira S., Sack D.A., Rashid M.U., Toslim Mahmud M., Mustafiz M., Rahman Z., Bhuyian S.I. (2016). Sustained uptake of a hospital-based handwashing with soap and water treatment intervention (cholera-hospital-based intervention for 7 days [CHoBI7]): A randomized controlled trial. Am. J. Trop. Med. Hyg..

[29] Eisenhauer I.F., Hoover C.M., Remais J.V., Monaghan A., Celada M., Carlton E.J. (2016). Estimating the risk of domestic water source contamination following precipitation events. Am. J. Trop. Med. Hyg..

[30] Exum N.G., Olórtegui M.P., Yori P.P., Davis M.F., Heaney C.D., Kosek M., Schwab K.J. (2016). Floors and Toilets: Association of Floors and Sanitation Practices with Fecal Contamination in Peruvian Amazon Peri-Urban Households. Environ. Sci. Technol..

[31] Heitzinger K., Rocha C.A., Quick R.E., Montano S.M., Tilley D.H., Mock C.N., Jannet Carrasco A., Cabrera R.M., Hawes S.E. (2015). Improved but not necessarily safe: An assessment of fecal contamination of household drinking water in rural Peru. Am. J. Trop. Med. Hyg..

[32] Reygadas F., Gruber J.S., Ray I., Nelson K.L. (2015). Field efficacy evaluation and post-treatment contamination risk assessment of an ultraviolet disinfection and safe storage system. Water Res..

[33] Benwic A., Kim E., Khema C., Phanna C., Sophary P., Cantwell R.E. (2018). Factors associated with post-treatment E. coli contamination in households practising water treatment: A study of rural Cambodia. Int. J. Environ. Health Res..

[34] Chik A.H.S., Schmidt P.J., Emelko M.B. (2018). Learning something from nothing: The critical importance of rethinking microbial non-detects. Front. Microbiol..

[35] El-Shaarawi A.H., Esterby S.R., Dutka B.J. (1981). Bacterial density in water determined by poisson or negative binomial distributions. Appl. Environ. Microbiol..

[36] Silvestri E.E., Yund C., Taft S., Bowling C.Y., Chappie D., Garrahan K., Brady-Roberts E., Stone H., Nichols T.L. (2017). Considerations for estimating microbial environmental data concentrations collected from a field setting. J. Expo. Sci. Environ. Epidemiol..

[37] CRD (2009). Systematic Reviews—CRD’s Guidance for Undertaking Reviews in Health Care.

[38] World Bank (2019). World Bank Analytical Classifications.

[39] Moher D., Liberati A., Tetzlaff J., Altman D.G. (2009). Preferred Reporting Items for Systematic Reviews and Meta-Analyses: The PRISMA Statement. PLoS Med..

[40] Institut National de la Statistique, UNICEF (2015). Enquête par Grappes à Indicateurs Multiples (MICS5 2014–2015), Rapport Final.

[41] Bangladesh Bureau of Statistics, UNICEF (2014). Bangladesh Multiple Indicator Cluster Survey 2012–2013, ProgotirPathey: Final Report.

[42] UNICEF (2016). Manual for Water Quality.

[43] R Core Team (2019). R: A Language and Environment for Statistical Computing.

[44] (1999). APHA/AWWA/WEF Part 9000 Microbiological Examination. Standard Methods for the Examination of Water and Wastewater.

[45] Chui K.K.H., Wenger J.B., Cohen S.A., Naumova E.N. (2011). Visual Analytics for Epidemiologists: Understanding the Interactions between Age, Time, and Disease with Multi-Panel Graphs. PLoS ONE.

[46] Pope M.L., Bussen M., Feige M.A., Shadix L., Gonder S., Rodgers C., Chambers Y., Pulz J., Miller K., Connell K. (2003). Assessment of the Effects of Holding Time and Temperature on *Escherichia coli* Densities in Surface Water Samples. Appl. Environ. Microbiol..

[47] Brown J., Stauber C., Murphy J.L., Khan A., Mu T., Elliott M., Sobsey M.D. (2011). Ambient-temperature incubation for the field detection of *Escherichia coli* in drinking water. J. Appl. Microbiol..

[48] Murray A.L., Kumpel E., Peletz R., Khush R.S., Lantagne D.S. (2018). The effect of sodium thiosulfate dechlorination on fecal indicator bacteria enumeration: Laboratory and field data. J. Water Health.

[49] Kulinkina A.V., Mohan V.R., Francis M.R., Kattula D., Sarkar R., Plummer J.D., Ward H., Kang G., Balraj V., Naumova E.N. (2016). Seasonality of water quality and diarrheal disease counts in urban and rural settings in south India. Sci. Rep..

[50] Alarcon Falconi T.M., Kulinkina A.V., Mohan V.R., Francis M.R., Kattula D., Sarkar R., Ward H., Kang G., Balraj V., Naumova E.N. (2017). Quantifying tap-to-household water quality deterioration in urban communities in Vellore, India: The impact of spatial assumptions. Int. J. Hyg. Environ. Health.

[51] Kaplan E.L., Meier P. (1958). Nonparametric Estimation from Incomplete Observations. J. Am. Stat. Assoc..

[52] El-Shaarawi A.H., Esterby S.R. (1992). Replacement of censored observations by a constant: An evaluation. Water Res..

[53] Helsel D.R. (2005). More than obvious: Better methods for interpreting nondetect data. Environ. Sci. Technol..

[54] Helsel D.R. (2006). Fabricating data: How substituting values for nondetects can ruin results, and what can be done about it. Chemosphere.

[55] Cha S.M., Lee S.W., Park Y.E., Cho K.H., Lee S., Kim J.H. (2010). Spatial and temporal variability of fecal indicator bacteria in an urban stream under different meteorological regimes. Water Sci. Technol..

[56] Ferguson A.S., Layton A.C., Mailloux B.J., Culligan P.J., Williams D.E., Smartt A.E., Sayler G.S., Feighery J., McKay L.D., Knappett P.S.K. (2012). Comparison of fecal indicators with pathogenic bacteria and rotavirus in groundwater. Sci. Total Environ..

[57] Korajkic A., McMinn B.R., Harwood V.J. (2018). Relationships between microbial indicators and pathogens in recreational water settings. Int. J. Environ. Res. Public Health.

[58] (2018). APHA/AWWA/WEF 9223 Enzyme Substrate Coliform Test. Standard Methods for the Examination of Water and Wastewater.

[59] Aquagenx Aquagenx ® CBT EC+TC (Compartment Bag Test) Most Probable Number (MPN) Kit Instructions for Use: Drinking Water.

[60] Michel M.C., Murphy T.J., Motulsky H.J. (2020). New author guidelines for displaying data and reporting data analysis and statistical methods in experimental biology. J. Pharmacol. Exp. Ther..

